# A Wheat WRKY Transcription Factor TaWRKY10 Confers Tolerance to Multiple Abiotic Stresses in Transgenic Tobacco

**DOI:** 10.1371/journal.pone.0065120

**Published:** 2013-06-10

**Authors:** Chen Wang, Pengyi Deng, Liulin Chen, Xiatian Wang, Hui Ma, Wei Hu, Ningcong Yao, Ying Feng, Ruihong Chai, Guangxiao Yang, Guangyuan He

**Affiliations:** The Genetic Engineering International Cooperation Base of Chinese Ministry of Science and Technology, Chinese National Center of Plant Gene Research (Wuhan) HUST Part, Key Laboratory of Molecular Biophysics of Chinese Ministry of Education, College of Life Science and Technology, Huazhong University of Science and Technology (HUST), Wuhan, China; University of Georgia, United States of America

## Abstract

WRKY transcription factors are reported to be involved in defense regulation, stress response and plant growth and development. However, the precise role of WRKY transcription factors in abiotic stress tolerance is not completely understood, especially in crops. In this study, we identified and cloned 10 WRKY genes from genome of wheat (*Triticum aestivum* L.). *TaWRKY10*, a gene induced by multiple stresses, was selected for further investigation. *TaWRKY10* was upregulated by treatment with polyethylene glycol, NaCl, cold and H_2_O_2_. Result of Southern blot indicates that the wheat genome contains three copies of *TaWRKY10*. The TaWRKY10 protein is localized in the nucleus and functions as a transcriptional activator. Overexpression of *TaWRKY10* in tobacco (*Nicotiana tabacum* L.) resulted in enhanced drought and salt stress tolerance, mainly demonstrated by the transgenic plants exhibiting of increased germination rate, root length, survival rate, and relative water content under these stress conditions. Further investigation showed that transgenic plants also retained higher proline and soluble sugar contents, and lower reactive oxygen species and malonaldehyde contents. Moreover, overexpression of the *TaWRKY10* regulated the expression of a series of stress related genes. Taken together, our results indicate that TaWRKY10 functions as a positive factor under drought and salt stresses by regulating the osmotic balance, ROS scavenging and transcription of stress related genes.

## Introduction

Environmental stresses, including drought, salinity and low temperature, are the primary causes of declines in crop yield and quality worldwide. To combat these challenges, plants have evolved sophisticated molecular networks resulting in adaptive responses through physiological and morphological changes [1]. The adaptive responses commonly use transcriptional activation or repression of genes upon signal perception and transduction of the external stimuli [2], [3]. Plant adaptability is mainly operated by the regulation of various transcription factors [4]. Significant progress has been made in understanding the genes response to various stresses [5], [6], [7], and numerous transcription factors and *cis*-regulatory sequences in plants have been identified [8]. Among them, the plant WRKY transcription factors, comprising a large family of regulatory proteins, are shown to play an important role in response to various stresses.

Since the first WRKY transcription factor was characterized from sweet potato (*Ipomoea batatas*) [9], many WRKY proteins have been identified from *Arabidopsis* (*Arabidopsis thaliana*), rice (*Oryza sativa*), soybean (*Glycine max*), barley (*Hordeum vulgare*), poplar (*Populus trichocarpa*), pinus (*Pinus monticola*) and *Physcomitrella patens*. The characteristic feature of the WRKY family is its highly conserved 60 amino acids, the WRKY domain, which is comprised of a highly conserved WRKYGQK motif at the N-terminus and certain zinc finger motifs at C-terminus [10]. The WRKYGQK motif in various plant species show slight variations in the amino sequences (for example, WRKYGKK and WRKYGEK) [11], [12]. Depending on their domain structures, the WRKY proteins can be divided into three different groups. Proteins with two WRKY domains belong to group I; proteins containing one WRKY domain belong to groups II or III, depending on the type of zinc finger motif [13], [14]. The WRKY factors present high binding affinity to a DNA *cis*-acting element named as the W box, (C/T)TGAC(T/C), which permits signal transduction to regulate the expressions of stress-related genes, resulting in plant stress tolerance finally [13].

During the process of plant evolution, the WRKY variations facilitate distinct cellular, developmental, and physiological roles. Accumulating evidence has demonstrated that the WRKY factors are central components of many aspects of the innate immune system of plants [15]. In addition, the WRKY factors participate in certain physiological processes, including embryogenesis [16], [17], seed development and germination [18], [19], trichome development [20], biosynthetic pathways regulation [21], [22] and hormone signaling [23], [24], [25]. Recently, attention has focused on biotic stress responsive WRKY transcription factors [21], [26], [27]. However, less progress has been made on understanding the function of WRKY proteins in the abiotic stress, as compared with the biotic stresses research progress [28].

Wheat is the dominant crop for human food and livestock feed. Current and future concerns include improving wheat yield and quality under hostile environments [29]. Considering the diverse roles of WRKY transcription factors under complex environmental conditions, clarifying the functions of certain WRKY members in the abiotic stress response remains a challenge. Overexpression of *WRKY* genes in *Arabidopsis* [30], rice [31] and soybean [32] conferred tolerance to abiotic stresses, especially oxidative stress [33], [34]. However, whether *WRKYs* confer drought and salt stress tolerance through reducing ROS accumulation is not yet to be determined in wheat. In the present study, based on the conserved protein sequence of WRKY transcription factors, expressed sequence tags (ESTs) with high similarity to the *WRKYs* in wheat genome sequence database were analyzed, collected and assembled into several unigenes. Ten of the *TaWRKYs*, designated *TaWRKY1*–*TaWRKY10*, were successfully identified. Among these genes, *TaWRKY10* was observed to confer drought and salt stress tolerance by regulating osmosis and reducing ROS accumulation.

## Materials and Methods

### Plant Materials and Stress Treatments

Wheat (*Triticum aestivum* L. cv. Chinese Spring) seeds were treated with 75% (v/v) ethanol for surface-sterilization and washed three times in distilled water. Seeds were germinated on distilled water and cultured in growth chambers (16 h light/8 h dark cycle with a light intensity of 200 µmol·m^−2^·s^−1^ at 25°C) for ten days. Polyethylene glycol (PEG) or NaCl treatment were conducted by transferring seedlings into Petri dishes containing 20% PEG6000 or 200 mM NaCl solutions, and incubated under light for 24 h. For cold treatment, seedlings were transferred to cold-chamber with a beaker containing water pre-cooled to 4°C and maintained at 4°C under light for 24 h. For treatment with signaling molecule, seedlings were sprayed with 10 mM H_2_O_2_, and incubated under light for 24 h. In all these treatments, wheat seedlings at similar growth states were used, and untreated wheat seedlings were taken as controls [35]. Leaf samples were frozen in liquid nitrogen, then stored at −80°C until RNA extraction. For tissue-specific expression analysis, the roots, stems and leaves of 10-day-old untreated seedlings were also collected.

### Cloning and Sequence Analysis of *TaWRKY10*

Total RNA from wheat leaves tissues was extracted using TRIzol reagent (Invitrogen, Carlsbad, CA, USA). After removing the genomic DNA contamination by DNase I (TaKaRa, Dalian, China), 200 ng Poly(A)^+^ mRNA was converted into cDNA by M-MLV Reverse Transcriptase (Promega, Madison, WI, USA). The cDNA template was used for PCR analysis subsequently [36]. We performed multiple alignment analysis using the wheat unigenes and ESTs are available on the DFCI wheat gene index database (http://compbio.dfci.harvard.edu/cgi-bin/tgi/gireport.pl?gudb=wheat) and wheat genome database (http://www.wheatgenome.org/). The full-length cDNA sequences were identified using DNAMAN software and amplified from wheat Poly (A)^+^ mRNA by PCR using specific primer pairs (see Table S1). After purification, the PCR products were combined with the pMD-18T plasmid (TaKaRa, Dalian, China) then sequenced. Domain prediction was performed by MEME (http://meme.sdsc.edu/meme/intro.html). This online software was used to create the logo representations of the WRKY domain and the rest of the alignment [37]. Multiple sequence alignment was performed by Clustal W [38] and Mega 4.0 [39]. Subcellular localization was predicted by Euk-mPLoc 2.0 (http://www.csbio.sjtu.edu.cn/bioinf/euk-multi-2/).

### Reverse Transcription-polymerase Chain Reaction (RT-PCR)

RT-PCR was used to determine the expression of specific ESTs in the WRKY gene family after treating wheat seedlings with 200 mM NaCl, cold (4°C) or 20% PEG6000. Primers (Table S1) used in RT-PCR had high specificity, as determined by agarose gel electrophoresis, and were also confirmed by sequencing. The RT-PCR reactions were performed using TaKaRa DNA polymerase for 30 cycles (TaKaRa, Dalian, China). Expression levels of target genes were normalized using *TaActin* as an internal control.

### Quantitative Real-time Polymerase Chain Reaction (qRT-PCR)

After RNA extraction and reverse transcription as described above, the resulting cDNA was used as the template for amplification with the MJ research Opticon detection system. The Opticon monitor qRT-PCR software was used for data analysis. The appearance of PCR products was monitored by detecting the increase of fluorescence caused by the binding of SYBR green dye (TOYOBO, Osaka, Japan) to dsDNA. The qRT-PCR was performed as described by Zhou *et al* [35]. The primer pairs’ efficiency and specificity were examined (Primers are provided in Table S1). In all experiments, each reaction had repeated at least three times and negative controls without templates were detected in case of contamination. The expression of *TaActin* or *NtUbiquitin* genes were used as the internal controls for normalization. The relative expression of mRNA was calculated using the 2^–ΔΔCt^ formula [40].

### Southern Blot Analysis

For analysis of wheat genome Southern blot, total wheat genomic DNA was extracted by CTAB method [41]. 10 µg genomic DNA was respectively treated with *Bam*HI, *Hin*dIII or *Sac*I restriction enzymes (MBI Fermentas), which cuts outside of the *TaWRKY10* coding sequence. For analysis of transgenic (TG) lines, the genomic DNA of TG tobacco lines was digested by *Hin*dIII. The *TaWRKY10* overexpressing vector was used as a positive control, and the genomic DNA of wild type (WT) tobacco was used as negative control. The digested genomic DNA was separated by electrophoresis and transferred to Hybond-N^+^ membrane by capillary blotting method. The membrane was hybridized with digoxigenin (DIG) labeled probe (Roche, Basel, Switzerland) (primer pairs are shown in Table S2) then detected by chemiluminescence method, according to Chen, *et al* [42].

### Subcellular Localization Assay

The *Nco*I and *Spe*I sites were added to the open reading frame (ORF) of *TaWRKY10* (Table S2), respectively. The gene was inserted into the pCAMBIA1304 vector containing the green fluorescent protein (GFP) gene and the *Cauliflower Mosaic Virus* (*CaMV*) 35 S promoter. The Subcellular localization of TaWRKY10-GFP fusion protein in onion (*Allium cepa* L.) epidermal cells was performed using the particle bombardment method (PDS-1000, Bio-Rad, Hercules, CA, USA), according to Hong, et al [43]. The GFP vector was used as a control. The DNA-specific nuclear stain 4',6-diamidino-2-phenylindole (DAPI) was used after bombardment [44]. Transformed cells were cultured for 24 h at room temperature in the dark on MS medium. Fluorescent microscopic images were collected using a fluorescence microscope (Karl Zeiss, Jena, Germany).

### Transcriptional Activation Assay

The binding specificity and transactivation activity of the TaWRKY10 protein were investigated in the yeast (*Saccharomyces cerevisiae*) strain AH109. The full length and deletions of *TaWRKY10* (WRKY, WRKY-N1, WRKY-N2, WRKY-C1 and WRKY-C2) were ligated to the yeast expression vector pGBKT7 (pBD) which having *His* and *LacZ* reporter genes (primers are provided in Table S2). The plasmid pBD was used as the negative control. These plasmids were transformed into yeast strains and were verified by PCR. The yeast strains were streaked on SD/−Trp and SD/−His plates containing 5 mM 3-amino-1, 2, 4-triazole (3-AT) and X-α-D-Galactoside (X-α-D-gal) [45], [46]. The plates were incubated at 30°C for 3 days.

### Anti-TaWRKY10 Polyclonal Antibody Preparation

The ORF of *TaWRKY10* was constructed into vector pET32a (Novagen, Billerica, MA, USA) to generate a fusion protein with a hexahistidine tag. The PCR product was digested with *Bam*HI and *Hin*dIII (Table S2). The identity of the recombinant pET32a-TaWRKY10 construct was confirmed by DNA sequencing and then transformed into *E. coli* BL21 (DE3) cells.

The BL21 cells were induced by 1 mM isopropyl-β-D-thiogalactoside (IPTG) for 4 h at 37°C. And then, the cells were harvested by centrifugation, and disrupted by physical fragmentation. Inclusion bodies were dissolved with 6 M urea on ice. The supernatant was filtered through a 0.45-µm membrane and purified by affinity chromatography using a nickel column (Ni-NTA agarose, Roche). The protein was renatured through step dialysis at 4°C for 12 h. The primary polyclonal antibody was produced *via* the immunization of TaWRKY10 protein in New Zealand rabbit [42].

### Western Blot Assay

The total proteins of tobacco overexpressing TaWRKY10 were extracted and separated by SDS-PAGE. After separation, proteins were transferred to a nitrocellulose membrane by electroblotted method. The membrane was blocked overnight at 4°C in TBST buffer with 5% nonfat milk. The membrane was incubated with primary rabbit anti-TaWRKY10 antibody (dilution 1∶10000 in TBST) at room temperature for 2 h. The membrane was washed with TBST buffer for three times and then incubated with 1∶10000 dilution of horseradish peroxidase-conjugated goat anti-rabbit IgG antibody for 1 h at 37°C (Santa Cruz Biotechnology, Santa Cruz, CA, USA). The *TaActin* was used as a control. The signal was scanned with Quantity One software (Bio-Rad).

### Generation of Transgenic Tobacco Plants

The tobacco transformation expression vector was constructed through the ligation of the *TaWRKY10* to the pSN1301 plasmid controlled by the CaMV 35 S promoter (Table S2). The pSN1301 was used as the vector control (VC). The construct was introduced into *Agrobacteria tumefaciens* strain LBA4404 competent cells by the freeze-thaw method [41]. Tobacco (*Nicotiana tabacum* L. cv. SR1) plants were transformed by the *Agrobacterium*-mediated leaf disc method [47]. The transgenic plants seeds were selected on MS medium [48] containing a final concentration of 40 mg/L of hygromycin. The regenerated seedlings were also confirmed by PCR. Three independent transgenic homozygous T_2_ line seedlings (Transgenic lines TG 1, TG 5 and TG 7) and the pSN1301 vector control line were used for subsequent experiments.

### Germination and Seedling Growth Assays

Approximately 100 seeds from each T_2_ generation tobacco plants of five independent lines WT, VC, TG 1, TG 5 and TG 7) were surface sterilized with 75% (v/v) ethanol and were sown on MS medium containing 100 mM NaCl, 100 mM mannitol or 5 mM H_2_O_2_. Plates were incubated in a 16 h light (25°C) and 8 h dark (20°C) chamber. The germination rate was scored daily for 7 days.

For early growth assessments, 5-day-old seedlings from vertical plates containing MS medium were placed with their roots pointing downwards onto vertically oriented plates with MS medium that were supplemented with NaCl (0, 50, 100, or 200 mM), mannitol (0, 50, 100, or 200 mM) or H_2_O_2_ (0, 1, 2 or 5 mM). Each plate containing WT, VC and TG lines, and five replicate plates were used for each treatment. Root lengths were marked at the onset of treatment, and their increases were monitored after 7 days [49].

### Drought and Salt Stress Treatments of Transgenic Tobacco Plants

One-month-old transgenic tobacco lines (TG 1, TG 5, TG 7 lines) and WT were subjected to different abiotic stress treatments. Thirty plants for each line were used for drought and salt stress treatment, respectively. For drought stress treatment, plants were withheld from watering for 3 weeks, and were then rewatered. Two weeks later, the rate of leaf yellowing and survival rate were calculated. For the salt stress treatment, plants were irrigated with 400 mM NaCl for 3 weeks, and then returned to the original growth conditions for 2 weeks. The rate of leaf yellowing and survival rate were calculated.

### Measurements of Relative Water, Free Proline, Soluble Carbohydrates and Malonaldehyde (MDA) Contents

Leaves of *TaWRKY10*-overexpression TG and WT tobacco plants were collected 3 weeks after each treatment for measurements. The relative water content (RWC) detection was performed as described by Zhou *et al* [35]. Proline content analysis was carried out by the ninhydrin reaction method. Fresh leaf material was extracted with sulfosalicylic acid, and the acetic acid and acid ninhydrin reagent were added into the solution and heated at 100°C for 30 min. After cooling to room temperature, the optical density of organic phase was determined at 520 nm. Soluble carbohydrate contents were determined by the phenol reaction method. Leaf tissue was boiled in water for extraction. After cooling to room temperature, the extract solution was mixed with 9% phenol and concentrated sulfuric acid. The tube was then shaken well and left to stand for 30 min. The aqueous extract was determined and recorded at 485 nm. The MDA content analysis was carried out by the thiobarbituric acid method. Fresh leaves were extracted with 10% trichloroacetic acid. After centrifugation at 4,000 g, supernatant was boiled with 0.5% thiobarbituric acid. Optical density readings of organic phase were taken at 532 nm, 600 nm and 450 nm and calculated as described by Draper *et al* [50].

### Detection of ROS

The detection of ROS assay was carried out according to the method described by Lee *et al*. [51]. Briefly, for superoxide (O_2_^−^) staining, tobacco leaves were treated with nitroblue tetrazolium (NBT) in HEPES buffer under vacuum infiltration. In the control treatment, MnCl_2_ and superoxide dismutase (SOD) were added to the system. For H_2_O_2_ staining, tobacco leaves were treated with 3, 3'-diaminobenzidine (DAB) in Tris-acetate buffer under vacuum infiltrated. The ascorbic acid was added to staining system as the control treatment. Samples were incubated over night at room temperature in the dark. After the staining, the tobacco leaves were bleached with 80% ethanol.

### Analysis of Downstream Genes Regulated by TaWRKY10

The WT and TG tobacco lines were cultured in soil under unstressed conditions for 30 days. Total RNA of the seedlings was extracted for reverse transcription to generate cDNA. Using quantitative PCR, the expression of stress related genes was detected. The *NtUbiquitin* gene was used as the internal reference. The sequences of the quantitative PCR primers are listed in Table S2.

### Statistical Analysis

Statistical analyses were carried out by Microsoft Excel and SPSS (Chicago, IL, USA). All the experiments were repeated for three times and Student’s *t*-test was applied for statistical analysis.

## Results

### The Characteristics of WRKYs in Wheat

**Figure 1:**
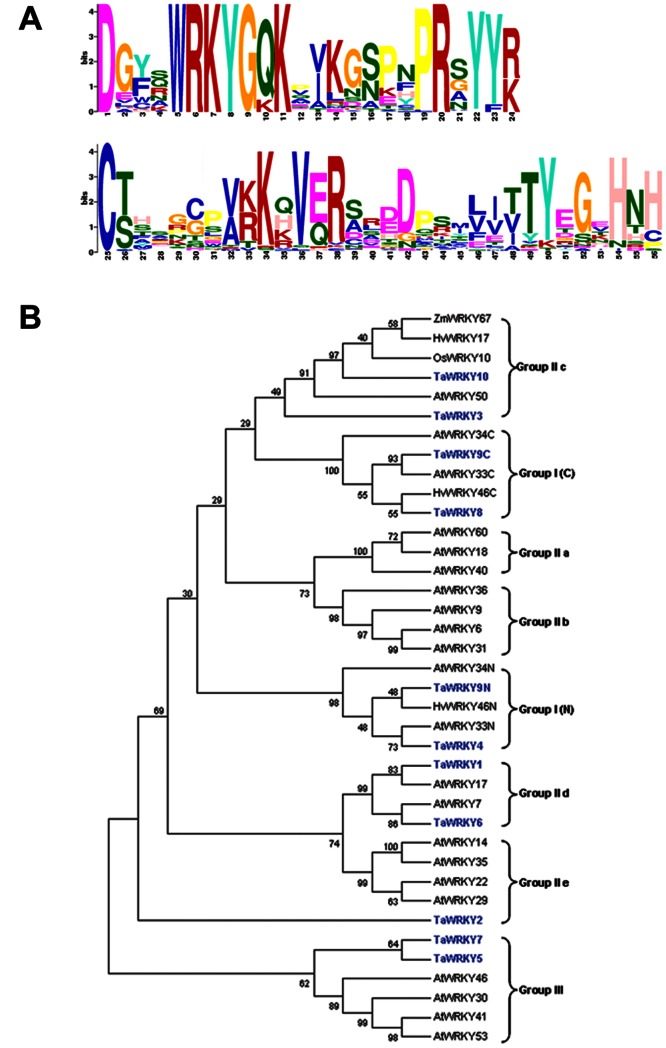
Predicted domains in the *TaWRKY1 - TaWRKY10* protein sequences and phylogenetic tree. (A) Predicted domains in the WRKY protein. The conserved domains were carried out by MEME using the protein sequences of TaWRKYs and other known WRKYs. This online software was used to create the logo representations of the WRKY domain and the zinc finger motif. On the y axis (measured in bits), depicts the overall height of the stack indicating the sequence conservation at that position, while the height of symbols within the stack indicates the relative frequency of each amino or nucleic acid at that position. (B) Phylogenetic tree of the TaWRKYs domains from various plants. The multiple alignments were generated by CLUSTAL W and the phylogenetic tree was constructed with MEGA4.0 using a bootstrap test of phylogeny with minimum evolution test and default parameters. GenBank accession numbers of WRKY proteins used for drawing phylogenetic tree are shown in Table S5.

To isolate WRKY genes from wheat, the WRKY conserved sequence was used as a query to search wheat ESTs in the DFCI database and the wheat genome database. After searching for WRKY domains and eliminating repeats, 10 WRKY transcription factors were identified from wheat leaves, of which nine possessed a complete ORF. They were designated *TaWRKY1*– *TaWRKY10*, respectively. The characteristics of WRKYs are provided in Table S3. Domain prediction of the full-length deduced proteins of the *TaWRKY*s clearly showed that these proteins contained the conserved WRKY DNA-binding domain and zinc finger region (Fig. 1A). These WRKYs were further divided into three subgroups: TaWRKY4, 8, and 9 belong to group I; TaWRKY1, 2, 3, 6, and 10 belong to subgroup II; and TaWRKY5 and 7 belong to group III (Fig. 1B). To elucidate the potential function of the 10 WRKY transcription factors in response to various stimuli, the expression patterns were analyzed by RT-PCR under various abiotic stress conditions (Table S4). Among the 10 *TaWRKY* genes, five WRKYs responded to at least one treatment. *TaWRKY10* was apparently induced by multiple treatments, and was chosen for further analysis.

The *TaWRKY10* cDNA is 791 bp in length (GenBank accession no. HQ700327), including a complete ORF of 672 bp encoding a putative protein of 223 amino acids (predicted relative molecular mass of 24.5 kDa). Analyzing the evolutionary relationships among the various species of WRKYs would provide an insight into the evolution of their function. To further characterize the TaWRKY10 protein, 34 WRKY domain proteins in different species were used for alignment. The alignment results revealed that the TaWRKY10 protein contains a conserved DNA-binding domain (WRKY domain) of 60 amino acids and a zinc finger region (C-X_4_-C-X_23_-H-X-H), indicating that it belongs to WRKY class II. TaWRKY10 is very similar to other WRKY proteins, such as *Phyllostachys edulis* (ADF42578.1) (85%), *Hordeum vulgare* (BAJ98268.1) (86%) and *Zea mays* (ACN29154.1) (85%). Based on the amino acid sequence alignment, two highly conserved regions were observed in TaWRKY10: four N-myristoylation sites and five Casein kinase II phosphorylation sites predicted by ExPASy Prosite analysis (Fig. S1). Protein myristoylation has been directly detected in *Arabidopsis*, rice, *Lycopersicon esculentum* and *Cucurbita pepo*, and is associated with proteins involved in growth regulation, disease resistance, salt tolerance and endocytosis [52]. These results suggested that *TaWRKY10* is a member of the WRKY family in wheat.

### Expression of *TaWRKY10* is Induced by PEG, NaCl, H_2_O_2_ and Cold Treatments

**Figure 2:**
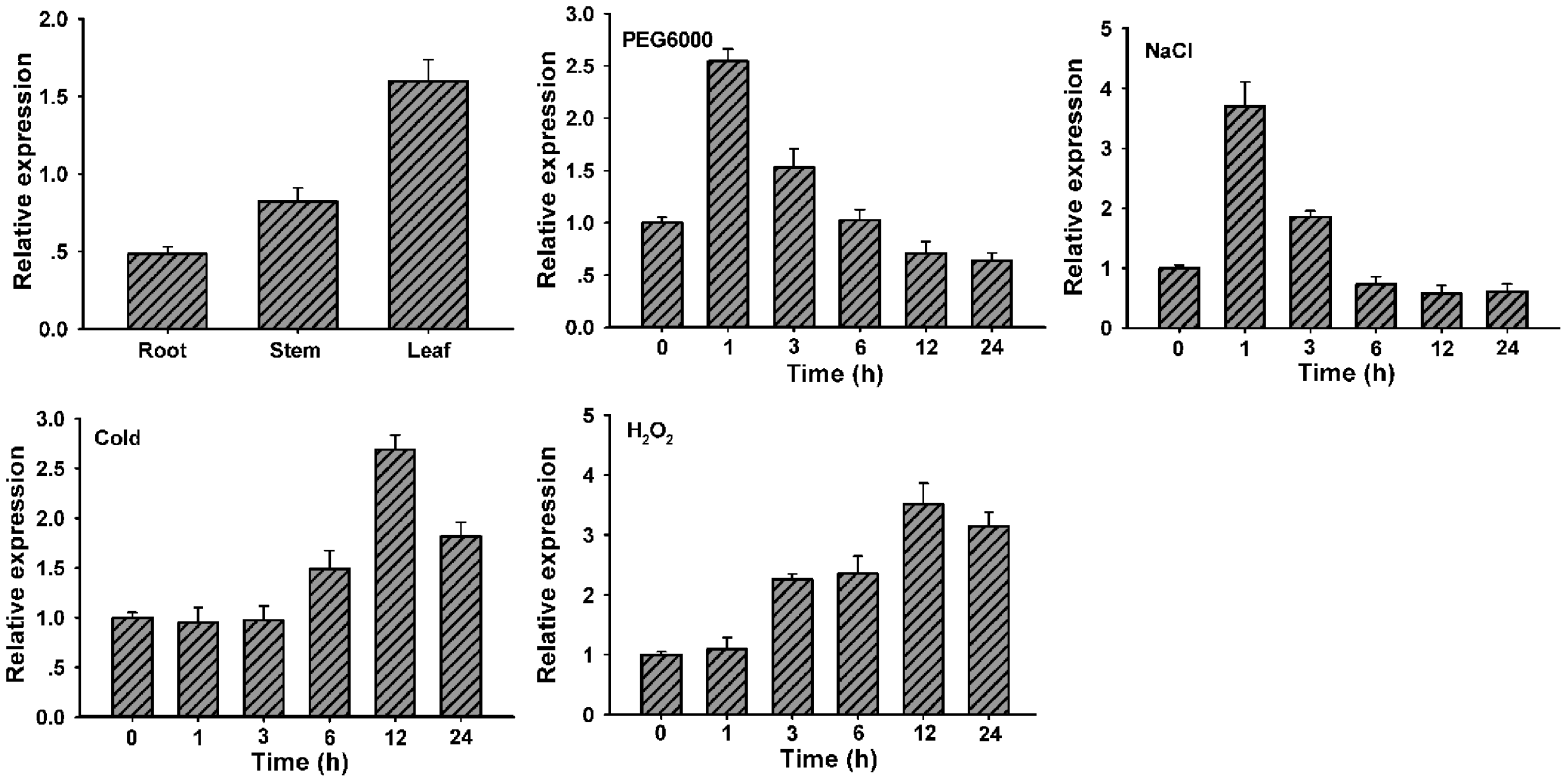
Expression patterns of wheat *TaWRKY10* in different tissues and under different treatments. The wheat seedlings were treated with 20% PEG6000, 200 mM NaCl, cold (4°C) or 10 mM H_2_O_2_. 200 ng Poly(A)^+^ mRNA was subjected to reverse transcription, and served as the qRT-PCR template. The y axis indicates the relative expression difference in mRNA level and the data were calculated using the 2^–ΔΔCt^ formula. The transcripts of *TaActin* in the same samples was using as a reference. Transcript levels of the *TaWRKY10* gene in untreated wheat were taken as 1. At least three biological experiments were carried out, which produced similar results.

To clarify the tissue expression patterns of *TaWRKY10*, mRNA isolated from different wheat tissues were using as the templates for qRT-PCR. *TaWRKY10* was detected to varying degrees of expression levels in root, stem and leaves of 10-day-old seedlings. To further characterize *TaWRKY10*, expression patterns of *TaWRKY10* under different abiotic stresses as well as signaling molecule were analyzed by qRT-PCR. As shown in Fig. 2, the *TaWRKY10* mRNA was induced and reached a maximum at 1 h after treatment with PEG6000 and NaCl. During cold treatment (4°C), *TaWRKY10* mRNA accumulated at 6 h after initiation of the treatment and peaked at 12 h. During H_2_O_2_ treatment, the expression of *TaWRKY10* was increased by 2.8-fold at 3 h and peaked at 12 h. These results suggested that the *TaWRKY10* gene is induced by PEG6000, NaCl, cold and H_2_O_2_ treatment.

### *TaWRKY10* is Present as Three Copies in Wheat Genome

**Figure 3:**
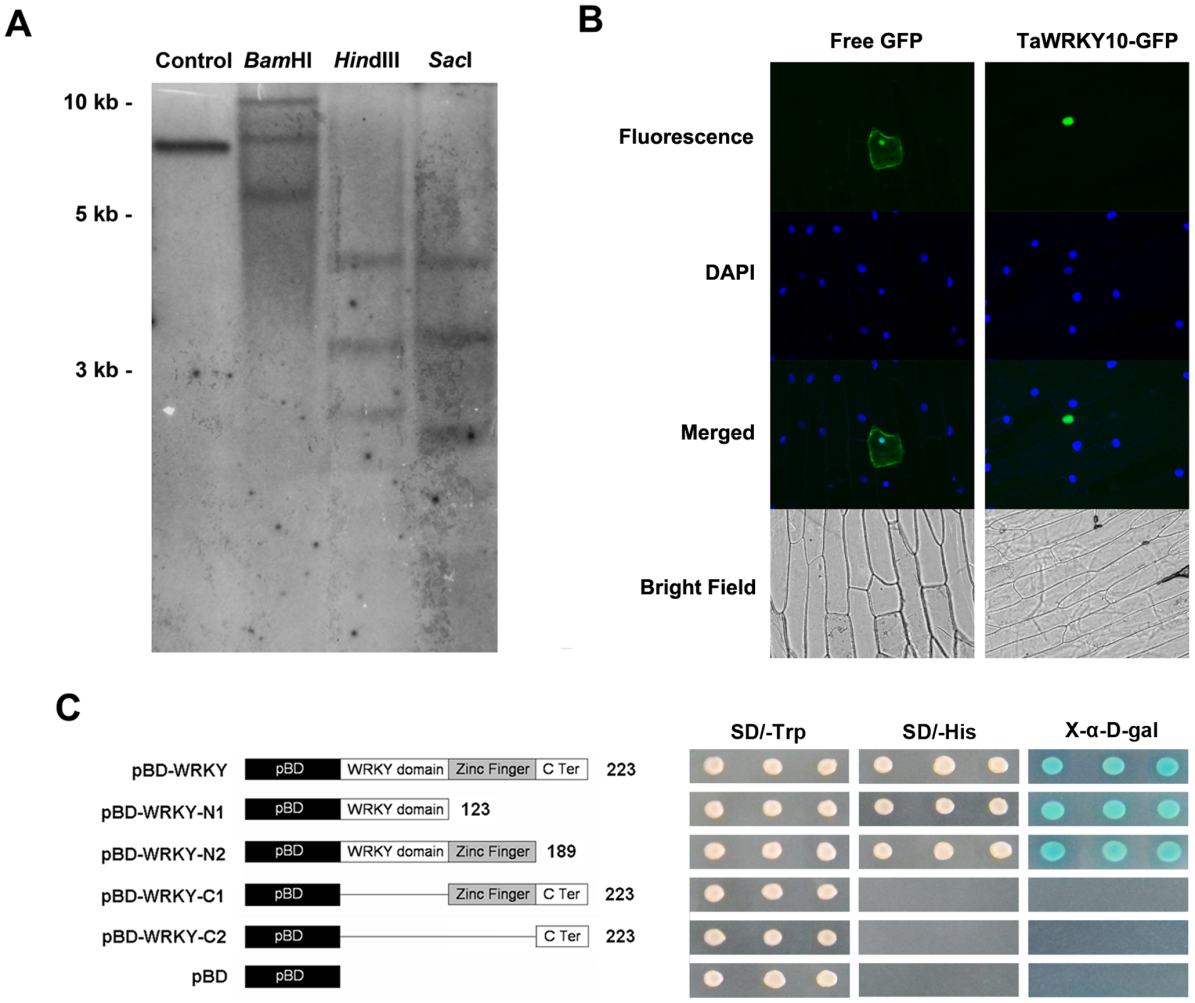
The genomic organization, protein localization and transcriptional activation activity of the *TaWRKY10* gene. (A) Southern blot analysis of the *TaWRKY10* gene. 10 µg genomic DNA of hexaploid wheat cv. Chinese Spring was digested completely with the restriction enzymes. The ORF of *TaWRKY10* was used as the hybrid probe. The *TaWRKY10* overexpressing vector was used as control. The 1 kb DNA Ladder (MBI Fermentas) are indicated on the left. (B) Subcellular localization of TaWRKY10 in onion epidermal cells. Onion epidermal cells were transferred with vector carrying GFP or TaWRKY10-GFP using bombardment method. Free GFP and TaWRKY10-GFP fusion proteins were transiently expressed in onion epidermal cells and observed with an inverted fluorescence microscope. (C) Transactivation activity of the TaWRKY10 protein in Yeast. The schematic diagram demonstrating the *TaWRKY10* cDNA fragments encoding different portions of TaWRKY10 that were fused to the yeast vector pGBKT7 (pBD). Transactivation activity analysis of TaWRKY10 was performed using yeast strain AH109. The transformants were streaked on the SD/−Trp or on SD/−His medium. The transformants were examined for growth in the presence of 3-AT and X-α-D-gal. Three biological experiments were carried out, which produced similar results.

To investigate the genomic architecture of *TaWRKY10* in hexaploid wheat, Southern blot assay of genomic DNA was performed. After high stringency washing, three hybridized bands were apparent in each lane. The result revealed that *TaWRKY10* was existed as three copies in the genomes of hexaploid wheat (Fig. 3A).

### The TaWRKY10 is Localized in the Nucleus

To characterize the subcellular localization of the TaWRKY10 protein, the TaWRKY10-GFP fusion protein was bombarded into onion epidermal cells. The subcellular localization of the TaWRKY10-GFP construct was observed *via* a fluorescence microscope. The nucleus location of TaWRKY10-GFP was confirmed by GFP and DAPI merging images showing a complete match (Fig. 3B). These results suggested that TaWRKY10 is a nuclear protein, possibly serving as a transcription factor.

### The TaWRKY10 Functions as a Potential Transcriptional Activator

To determine the *TaWRKY10* transcriptional activation activity in eukaryotic cells, a yeast expression system was used. The ORF of *TaWRKY10* gene was combined with the DNA-binding domain to identify transcriptional activation activity. The constructs pBD-WRKY, pBD-WRKY-N1, pBD-WRKY-N2, pBD-WRKY-C1 and pBD-WRKY-C2 were transformed into yeast strain AH109, and pBD was used as a control. As shown in Fig. 3C, the yeast cells transformed with pBD-WRKY pBD-WRKY-N1 and pBD-WRKY-N2 grew well in the SD/−His medium and SD/−Trp medium. Meanwhile, yeast cells transformed with pBD-WRKY-C1, pBD-WRKY-C2 and pBD could only survive in the SD/−Trp medium. The staining result showed that the yeast cells turned blue in the presents of X-α-D-gal. The results indicated that the *His* and *LacZ* reporter genes were activated and the presence of transcriptional activity in the full-length TaWRKY10 protein and N-terminal domain.

### Transgenic Tobacco Plants Obtained

**Figure 4:**
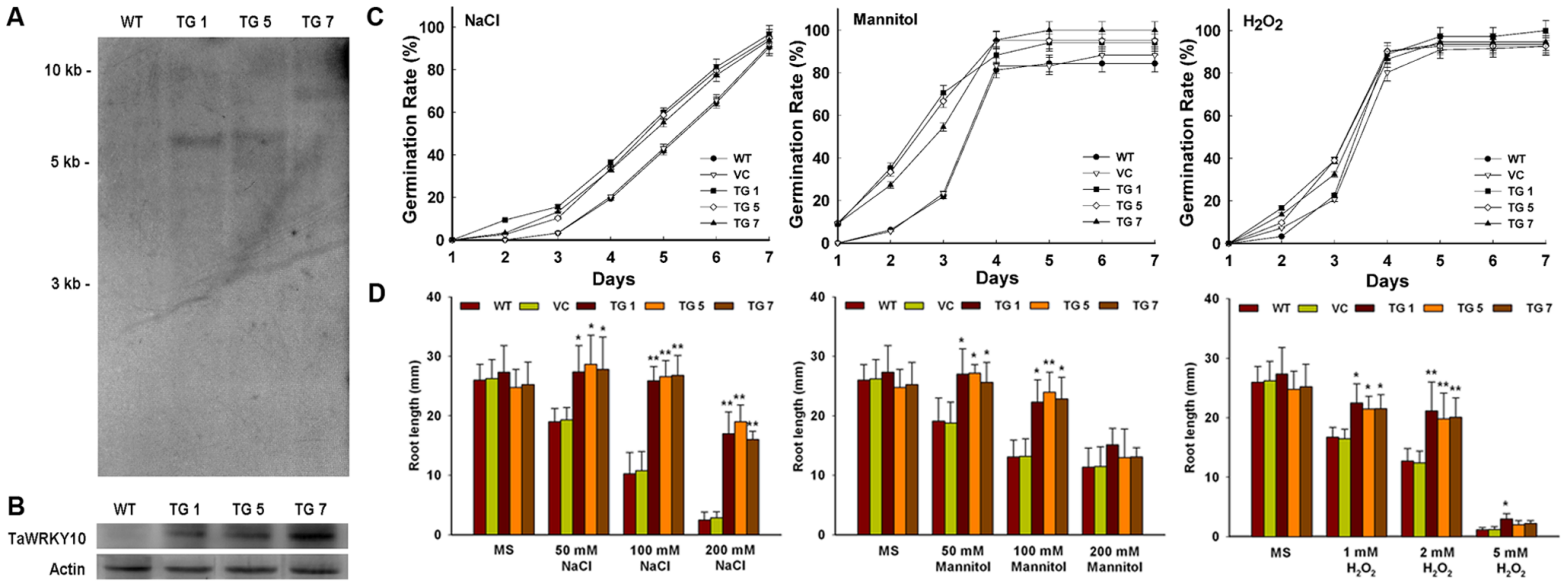
Identification and the early development assay of the *TaWRKY10* transformed tobacco plants. (A) Southern blot confirmation of the *TaWRKY10* copy number. The 1 kb DNA Ladder (MBI Fermentas) is indicated on the left. (B) Western blot confirmation of TaWRKY10 protein expression. (C) Germination rate of tobacco overexpressing the *TaWRKY10* gene. (D) Root lengths of tobacco plants overexpressing the *TaWRKY10* gene. The data present means ± SD of three experiments performed. Significant differences between the TG and control lines are indicated as *p<0.05; **p<0.01.

The full-length *TaWRKY10* sequence was transformed into WT tobacco line. Seventeen independent TaWRKY10 overexpressing TG lines were obtained and confirmed by genomic PCR. Among the T_1_ lines, three independent lines (TG 1, TG 5 and TG 7) segregated at a 3∶1 ratio on hygromycin resistance. Moreover, seedlings from all three transgenic T_2_ lines grew well on MS medium supplemented with hygromycin. Among the T_2_ lines, the Southern blot assay result showed one copy of the *TaWRKY10* was integrated into the genomes of transgenic lines TG 1, TG5 and TG 7 (Fig. 4A). Those transgenic lines showed high expression of the TaWRKY10 protein, as confirmed by Western blotting (Fig. 4B). The results indicated that *TaWRKY10* was present as a single copy and was stably inherited in T_1_–T_2_ transgenic lines. Three independent T_2_ lines of the *TaWRKY10* transgenic plants were chosen for further analysis.

### Overexpression of *TaWRKY10* Enhances Tolerance to Mannitol, NaCl and H_2_O_2_ Treatments during Seed Germination and Root Elongation

To evaluate the stress tolerance of the control and the TG tobacco lines, seed germination was examined. Seeds were sown on MS medium supplemented with appropriate concentrations of mannitol, NaCl or H_2_O_2_. Germination rates of WT seeds, VC seeds and TG seeds did not show any significant difference on normal MS medium (data not shown). As shown in Fig. 4C, when seeds were germinated on 100 mM mannitol for 3 days, only 21.9% germination was seen in the WT line and 23.3% in the VC line, whereas the TG lines showed 50%–70% germination. After 7 days of germination, only 84.3% of WT and 88.2% of VC seeds germinated, whereas at least 94.1% germination was observed in the three TG lines. Under mannitol stress, the percentage germination of WT was much lower compared with that of the TG over a 7-day period. When the seeds of WT, VC and TG lines were germinated in the presence of 100 mM NaCl, the TG lines germination rates (TG 1–96.8%, TG 5–94.9% and TG 7–93.8%) were higher than those of WT plants (90.5%) and VC plants (91.5%). When the seeds were germinated on MS medium containing 5 mM H_2_O_2_ for 2 days, the germination rates of TG seeds were significantly higher than the WT line and VC line. Only 22.6% of WT seeds and 20.1% of VC seeds germinated at day 2, compared with 38.9%, 39.0% and 32.4% of seeds hosting the *TaWRKY10* gene.

Subsequently, root elongation was tested under gradient NaCl, mannitol or H_2_O_2_ treatments (Fig. S2). As the concentration of NaCl increased, the root lengths of the control lines were significantly arrested, whereas the TG roots continued to grow. Under mannitol and H_2_O_2_ treatments, the TG plants also showed more adaptation to stress compared with the control lines. As shown in Fig. 4D, the growth situation displayed little difference between the WT line and VC line grown on different medium. Thus, these results indicated that overexpression of *TaWRKY10* in tobacco increased tolerance to mannitol, NaCl and H_2_O_2_ stresses during seed germination and root elongation.

### Overexpression of *TaWRKY10* Enhances Drought and Salt Tolerance in Transgenic Tobacco Plants

**Figure 5:**
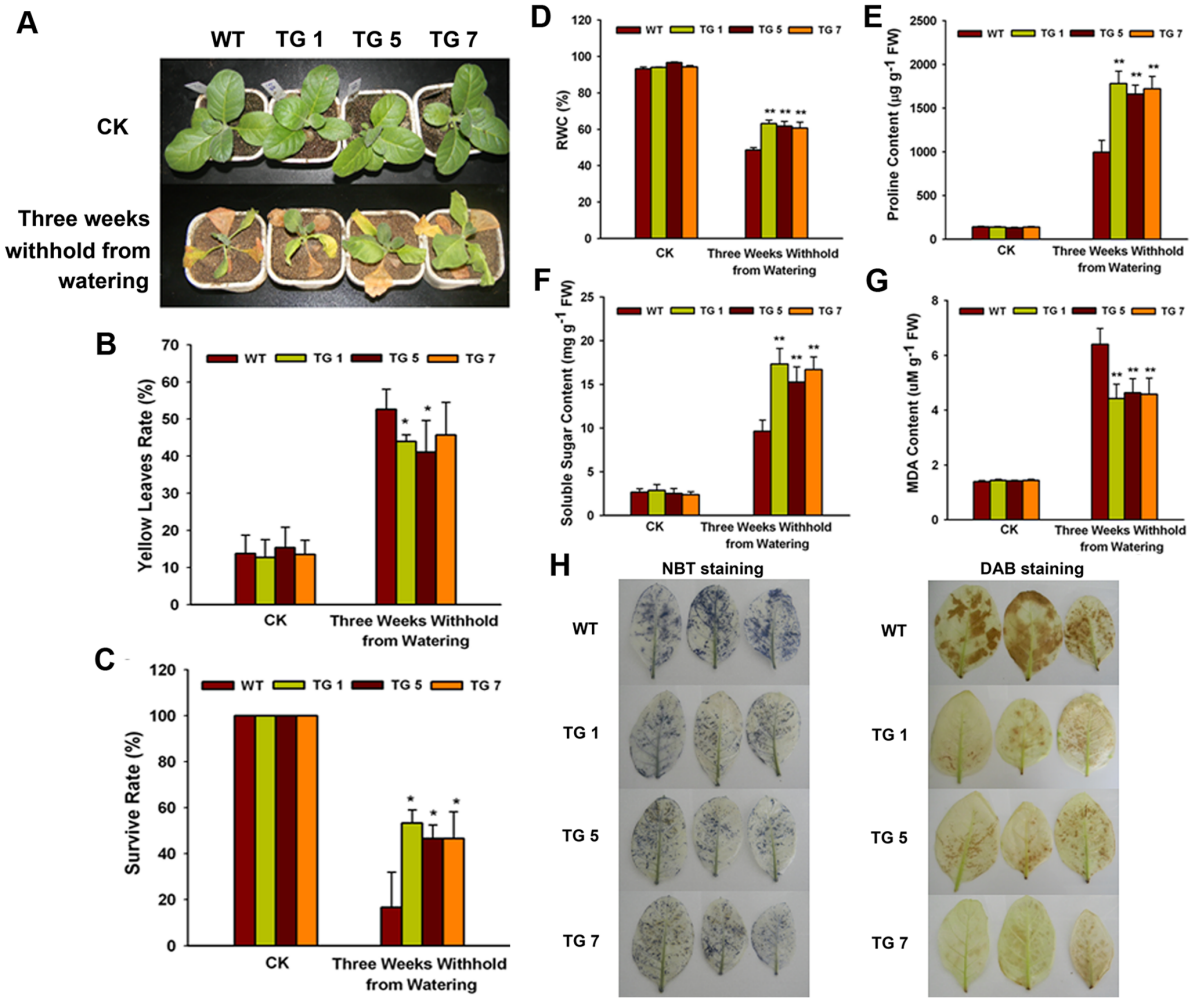
Analysis of the enhanced drought tolerance in transgenic tobacco lines. (A) Phenotype of WT and TG tobacco lines after 3 weeks of drought treatment. (B) Rate of leaf yellowing. (C) Survival rate. (D) RWC content. (E) Proline content. (F) Soluble sugar content. (G) MDA content. (H) Tissue localization of O_2_^−^ generation by NBT staining and tissue localization of H_2_O_2_ generation by DAB staining. The data present means ± SD of three experiments performed. Significant differences between the TG and control lines are indicated as *p<0.05; **p<0.01.

**Figure 6:**
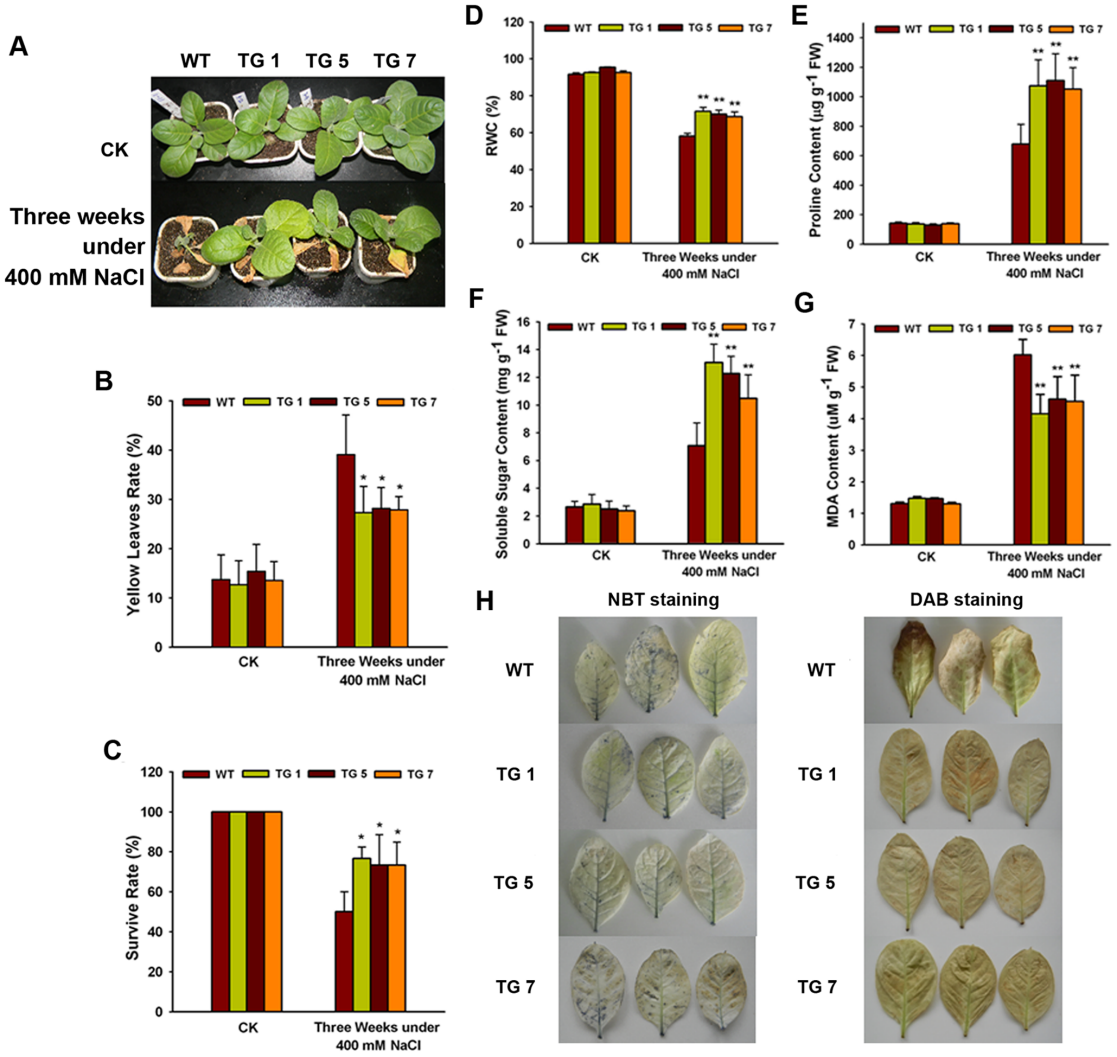
Analysis of the enhanced salt tolerance in transgenic tobacco lines. (A) Phenotype of WT and TG tobacco after 3 weeks of 400 mM NaCl treatment; (B) Rate of leaf yellowing. (C) Survival rate. (D) RWC content. (E) Proline content. (F) Soluble sugar content. (G) MDA content. (H) Tissue localization of O_2_^−^ generation by NBT staining and tissue localization of H_2_O_2_ generation by DAB staining. The data present means ± SD of three experiments performed. Significant differences between the TG and control lines are indicated as *p<0.05; **p<0.01.

To characterize the performance of *TaWRKY10* transgenic lines under drought and salt stress in soil, all three TG lines, and the controls, were tested at the seedling stage. Comparing with the control plants under normal conditions, the TG tobacco plants showed no obvious phenotypic difference in terms of appearance, flowering time or production. However, under stress conditions, the TG plants did show differences in performance. After drought treatment for 3 weeks, compared with TG plants, the WT plants were smaller and more withered (Fig. 5A). After 3 weeks of exposure to 400 mM NaCl, most leaves of the TG tobacco remained green, while leaves of the WT turned yellow (Fig. 6A). The TG lines exhibited lower rates of leaf yellowing (Fig. 5B and 5C) and higher survival rates (Fig. 6B and 6C) than WT lines under drought and salt treatments. The rate of leaf yellowing, plant height and survival rate are the typical phenotypic and physiological parameters used to evaluate resistance in crop plants. Plants that are taller, with higher survival rates and fewer yellow leaves are more tolerant to stresses. The phenotypic characterization suggested that overexpression of *TaWRKY10* enhanced drought and salt stress tolerance.

### Overexpression of the *TaWRKY10* Increases RWC, Proline and Sugar Accumulation, and Decreases MDA and ROS under Drought and Salt Stresses

To investigate the physiological differences between control and TG plants, some important physiological indices were measured. Compared with the WT plants, the TG lines showed remarkably higher levels of RWC (Fig. 5D and 6D), proline (Fig. 5E and 6E) and soluble sugar (Fig. 5F and 6F), but lower levels of MDA (Fig. 5G and 6G) under drought and salt conditions. Subsequently, the presence of ROS in WT lines and TG lines was detected with NBT staining and DAB staining. Under normal conditions, both WT and TG seedlings accumulated less superoxide. Moreover, no staining was detected in WT or TG lines in control treatment. The control treatment result suggested that the staining was caused by superoxide or hydrogen peroxide specifically. After exposure to drought or salt treatments, the ROS level of WT plants accumulated greater than TG seedlings (Figs. 5H and 6H). DAB staining showed that the WT line accumulated more hydrogen peroxide along the main vein under drought and salt stresses. The staining results suggesting that overexpression of *TaWRKY10* decreased the accumulation of ROS under drought and salt treatments. In conclusion, the physiological characterization results suggested that overexpression of *TaWRKY10* increased RWC, proline and sugar content, and decreased MDA and ROS accumulation under drought and salt conditions.

### Overexpression of the *TaWRKY10* Significantly Activates the Expression of Stress Related Genes in Tobacco Plants

**Figure 7:**
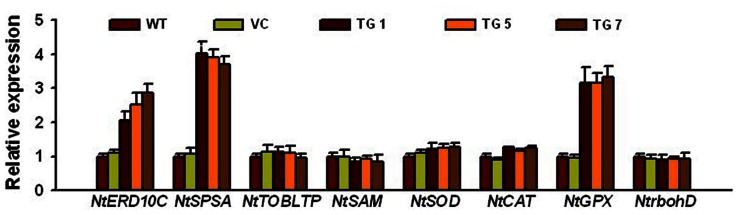
Overexpression of the *TaWRKY10* gene in tobacco enhances the expression of stress related genes. The y axis indicates the relative expression difference in mRNA levels of these genes in TG tobacco, and the data were calculated using the 2^–ΔΔCt^ formula. The transcripts of *NtUbiquitin* in the same samples was using as a reference. Three biological experiments were performed, which produced similar results.

To gain a deeper understanding of the function of *TaWRKY10* during drought and salt stress, we detected the expression of eight osmotic stress related genes in the control and TG lines by qRT-PCR. The expression of three genes in overexpressed seedlings was obviously higher in TG lines than in control plants (Fig. 7). Genes selected for this analysis include *NtERD10C* and *NtSPSA* related to osmotic stress, and *NtGPX* (encoding glutathione peroxidase) involved in scavenging ROS, suggesting that *TaWRKY10* constitutively induced the expression of osmotic stress and oxidative stress genes in tobacco plants.

## Discussion

Recently, more attention has been paid to WRKY transcription factors in regulating plant growth and development and biotic stress; however, their precise functions remain uncertain. Moreover, compared with the progress on biotic stresses, less progress has been made on understanding the function of WRKY proteins in the abiotic stress [25]. Wheat is the foremost staple food crop in the world which provides both calories and proteins to over 35% of the human population [53]. The production of wheat is affected by multiple environmental stresses, including drought, salinity and extreme temperatures. However, only two wheat WRKYs have been characterized; *TaWRKY2* and *TaWRKY19* are involved in abiotic stresses responses [28]. In the present study, 10 non-redundant wheat WRKYs were identified and *TaWRKY10* was characterized to function as a positive factor in abiotic stresses.

### *TaWRKY10* is a Member of the WRKY Family in Wheat

The conserved primary structural features of the TaWRKYs were identified using MEME (Fig. 1a). All 10 deduced WRKY proteins have WRKY domains and zinc finger motifs. Notably, TaWRKY4 and TaWRKY8, which carry only one WRKY domain, belong to group I WRKY proteins (Fig. 1b). This might be explained by the secondary loss of an N-terminal WRKY domain [13]. The result of subcellular localization of TaWRKY10-GFP indicated that the construct was located in the nucleus (Fig. 3B), which is consistent with previous studies on WRKY transcription factors from other species [54]. The localization data were consistent with the results of bioinformatics analysis, which showed that TaWRKY10 contained a nuclear localization signal. This implies that it acts as part of a transcription-regulating complex, as predicted by Euk-mPLoc 2.0 (data not shown). Transcriptional activation analysis showed that the transcriptional activity in the presence of full-length TaWRKY10 protein and N-terminal domain. Activating regions are typically acidic and probably form part of an α-helix. The N-terminal region of *TaWRKY10* is acidic (amino acid 31–70, pI  = 3.62), which has transcriptional activity. These data conclude that the *TaWRKY10* is a member of the WRKY family in wheat and may serve as a transcription activator.

### TaWRKY10 Plays a Positive Role during Abiotic Stresses

In our study, quantitative PCR demonstrated that *TaWRKY10* was induced by multiple stresses, including PEG6000, NaCl, cold (4°C) and H_2_O_2_ (Fig. 2). Responses to abiotic stimuli of WRKY transcription factors are often extremely rapid and transient. The WRKY transcription factors mediate signals transduction *via* activating adaptive responses and regulation of downstream genes. During responses to multiple stresses, a single WRKY gene often participates various signaling pathways, indicating its diverse regulatory mechanism. The expressions of certain stress-induced genes have been demonstrated to be associated with stress tolerance [25]. The response of *TaWRKY10* to a broad range of environmental stresses implied that *TaWRKY10* might be involved in different stress signaling pathways as a connection point.

The *in vivo* role of *TaWRKY10* in plant resistance was clearly demonstrated in transgenic tobacco lines transformed with an overexpression construct for *TaWRKY10*. Under drought and salt stress conditions, the WT plants were smaller, more withered and more yellow, while the TG tobaccos were more able to adapt to those stresses (Figs. 5 and 6). The rate of leaf yellowing and the survival rate are typical phenotypic and physiological parameters used for evaluating plants resistance. Plants with higher survival rate and fewer yellow leave have higher tolerance and are more resistant to stresses. Our findings were consistent with previous results reporting that *BcWRKY46*, *TaWRKY2*, *TaWRKY19*, and *HvWRKY38* conferred drought and salt stresses, and enhanced stress tolerance, in transgenic plants [28], [55], [56]. Data showed that the overexpression of *TaWRKY10* in tobacco led to adaptation to drought and salt stresses.

### Overexpression of the *TaWRKY10* in Tobacco Increases Drought and Salt Resistance

After drought or salt treatment for 3 weeks, TG lines accumulated higher levels of RWC, proline and soluble sugar (Figs. 5 and 6). RWC is a relevant index for measuring plant water status under drought tolerance. Plants accumulate several metabolites, such as proline, and a variety of sugars and sugar alcohols to prevent these detrimental changes [57]. The accumulations of free proline and soluble sugar in plants play highly protective roles under stresses conditions. Studies have shown that plants with higher proline and soluble sugar had better stress resistances [58]. Thus, our results indicated that the TG lines increase drought and salt resistance by osmoregulation.

It has been reported that abiotic stress causes lipid peroxidation, leading to MDA accumulation [59], [60], [61]. MDA content could be used as a measure of the damage caused by abiotic stresses [62]. Our results demonstrated that TaWRKY10 might protect the plant by decreasing the accumulation of MDA. In the second experiment, *TaWRKY10*-overexpressing plants exhibited lower O_2−_ and H_2_O_2_ accumulation than WT plants under drought and salt stress conditions (Figs. 5H and 6H). ROS, which comprise O_2−_, H_2_O_2_, singlet oxygen (^1^O_2_) and hydroxyl radicals (HO·), are highly reactive and toxic to cells, and finally result in oxidative damages [63]. Plants have to maintain their ROS balance to minimize cellular damage caused by stress. Among the variety of ROS compounds, the present study investigated the effect of superoxide (O_2−_) and hydrogen peroxide (H_2_O_2_) in abiotic stress resistant. The results showed that overexpression of *TaWRKY10* reduced cellular injuries caused by ROS in TG seedlings. Consequently, we infer that overexpression of *TaWRKY10* in tobacco confers drought and salt stress tolerance by reducing ROS accumulation.

### TaWRKY10 Confers Drought and Salt Stress through Stress Related Genes

To gain a further insight into the function of *TaWRKY10* in stress tolerance at the molecular level, the expressions of 8 stress related genes were investigated (Fig. 7). *NtERD10C* belongs to LEA-like proteins, which are highly hydrophilic and glycine-rich [64]. The LEA-like proteins pervade throughout all organisms and can be induced by osmotic stress suggests that LEA-like proteins may acclimatize to osmotic stress [65], [66], [67]. *SPSA* is critical in the synthesis of sucrose in photosynthetic and non-photosynthetic tissues. Diverse choices for posttranslational modification of the SPSA protein allow enzyme activity adaptate to severe environment rapidly [68]. This is especially important in actively photosynthesizing leaves during adaptation to salt stress [69]. The results in this study suggested that *TaWRKY10* constitutively regulated the expression of genes that involved in osmoregulation in TG seedlings. Subsequently, we examined the expression of genes response to oxidative stress. GPXs are enzymes that catalyze decreases in hydrogen peroxide, organic hydroperoxide and lipid hydroperoxide, and protect cells against oxidative radicals [70]. Plant GPX proteins or transcripts increased in response to several stresses, including biotic and abiotic stresses [71]. Our results indicated that *TaWRKY10* alters the expression of oxidative related genes in response to stress. Thus, it might be that abiotic stresses regulate the expression of *TaWRKY10*; the accumulation of the TaWRKY10 then most likely upregulates the transcription of downstream stress-inducible genes, and the obtained proteins resulted in the increased the resistance to stress conditions.

In conclusion, our results clearly demonstrated that TaWRKY10 is a stress-inducible wheat transcription factor, and that the *TaWRKY10* was up-regulated by PEG, NaCl, cold and H_2_O_2_. *TaWRKY10* could enhance drought and salt stress tolerances in transgenic tobacco plants. These functions are achieved by accumulating water, proline and soluble sugar contents, and by reducing plant ROS and MDA contents. *TaWRKY10* confers drought and salt stress tolerance by regulating the expression of stress related genes, thus protecting plants from damage. However, further evidences are needed to verify whether these genes are direct targets of TaWRKY10s. Thus, exploring the specific and direct genes regulated by TaWRKY10, together with its interacting partners will reveal the exact mechanisms of plant responses to abiotic stresses.

## Supporting Information

Figure S1 **Sequence analysis of the TaWRKY10 protein.** Boxes represent casein kinase II phosphorylation sites. Sequences with single underlined indicate N-myristoylation sites, sequence marked with double underlines refer to WRKY domain. Zinc-finger motif is marked with asterisk.
(DOC)

Figure S2 **Root lengths of tobacco plants overexpressing the** ***TaWRKY10*** **gene under different stress conditions.** The WT, VC and TG lines were cultured in MS medium under a 16 h light/8 h dark cycle at 25°C for 1 week, and then the seedlings were transplanted to fresh MS medium or MS medium supplied with 100 mM NaCl or 100 mM Mannitol or 2 mM H_2_O_2_ for 1 week. Then the photographs were taken. Three biological experiments were carried out, which produced similar results.
(DOC)

Table S1 **Gene specific primers used for isolating wheat WRKY genes and RT-PCR analysis.**
(DOC)

Table S2 **Primers for** ***TaWRKY10*** **used in this article.**
(DOC)

Table S3 **Characteristics of WRKYs from wheat.**
(DOC)

Table S4 ***TaWRKY1*** **-** ***TaWRKY10*** **expression patterns in wheat (** ***Triticicum aestivum*** **cv Chinese Spring) under abiotic stresses.**
(DOC)

Table S5 **The GenBank accession numbers of WRKY proteins used for drawing phylogenetic tree.**
(DOC)
