# Supplementary material for: A Wheat WRKY Transcription Factor TaWRKY10 Confers Tolerance to Multiple Abiotic Stresses in Transgenic Tobacco
Source: PLoS One. 2013 Jun 10;8(6):e65120. doi: 10.1371/journal.pone.0065120 (PMC3677898; doi:10.1371/journal.pone.0065120)
Supplement: Figure S1 — Sequence analysis of the TaWRKY10 protein. Boxes represent casein kinase II phosphorylation sites. Sequences with single underlined indicate N-myristoylation sites, sequence marked with double underlines refer to WRKY domain. Zinc-finger motif is marked with asterisk. (DOC) [file pone.0065120.s001.doc]

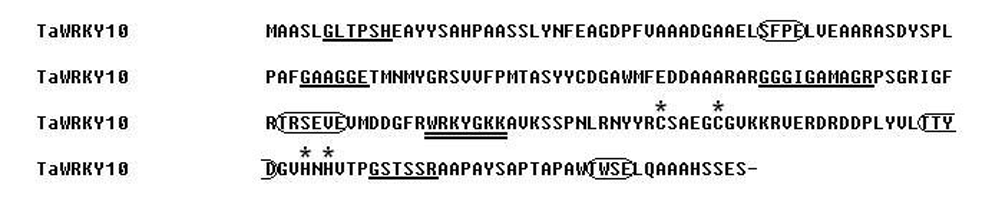


**Fig. S1 Sequence analysis of the TaWRKY10 protein.** Boxes represent casein kinase II phosphorylation sites. Sequences with single underlined indicate N-myristoylation sites, sequence marked with double underlines refer to WRKY domain. Zinc-finger motif is marked with asterisk.
