# Supplementary material for: A Wheat WRKY Transcription Factor TaWRKY10 Confers Tolerance to Multiple Abiotic Stresses in Transgenic Tobacco
Source: PLoS One. 2013 Jun 10;8(6):e65120. doi: 10.1371/journal.pone.0065120 (PMC3677898; doi:10.1371/journal.pone.0065120)
Supplement: Figure S2 — Root lengths of tobacco plants overexpressing the TaWRKY10 gene under different stress conditions. The WT, VC and TG lines were cultured in MS medium under a 16 h light/8 h dark cycle at 25°C for 1 week, and then the seedlings were transplanted to fresh MS medium or MS medium supplied with 100 mM NaCl or 100 mM Mannitol or 2 mM H2O2 for 1 week. Then the photographs were taken. Three biological experiments were carried out, which produced similar results. (DOC) [file pone.0065120.s002.doc]

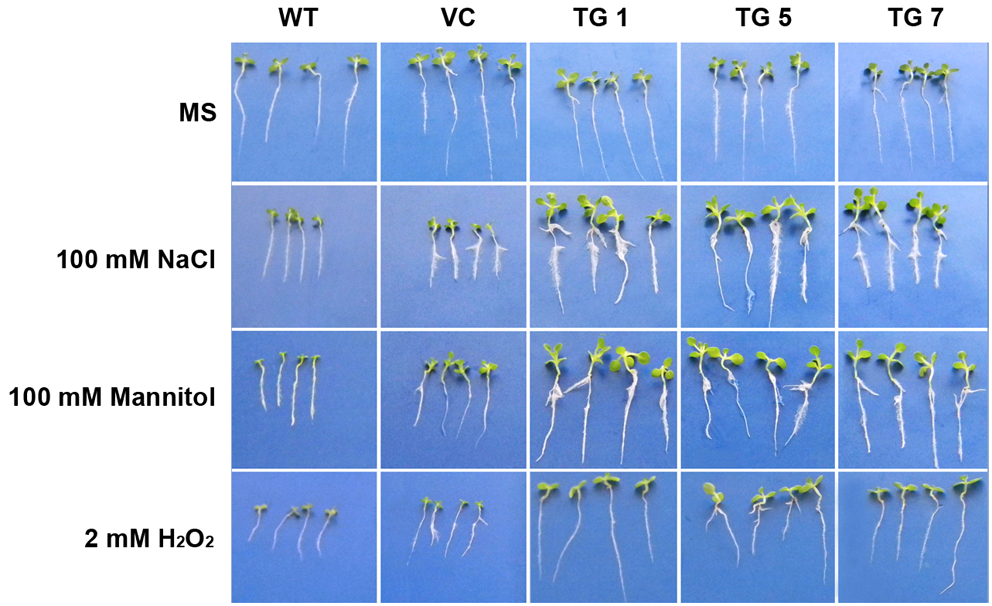


**Fig. S2 Root lengths of tobacco plants overexpressing the *TaWRKY10* gene under different stress conditions.** The WT, VC and TG lines were cultured in MS medium under a 16 h light/8 h dark cycle at 25 °C for 1 week, and then the seedlings were transplanted to fresh MS medium or MS medium supplied with 100 mM NaCl or 100 mM Mannitol or 2 mM H_2_O_2_ for 1 week. Then the photographs were taken. Three biological experiments were carried out, which produced similar results.
