# Supplementary material for: A Wheat WRKY Transcription Factor TaWRKY10 Confers Tolerance to Multiple Abiotic Stresses in Transgenic Tobacco
Source: PLoS One. 2013 Jun 10;8(6):e65120. doi: 10.1371/journal.pone.0065120 (PMC3677898; doi:10.1371/journal.pone.0065120)
Supplement: Table S1 — Gene specific primers used for isolating wheat WRKY genes and RT-PCR analysis. (DOC) [file pone.0065120.s003.doc]

**Table S1 Gene specific primers used for isolating wheat WRKY genes and RT-PCR analysis.**

| Gene Name | Primer Name | Sequences of 5'- and 3'- Primers |
| --- | --- | --- |
| *TaWRKY1* | *TaWRKY1* Full Length | 5'- GACCACACACCGGCGACCAT -3'/5'- ACTAAGTCAGACGTGCCCGTTGC -3' |
| *TaWRKY2* | *TaWRKY2* Full Length | 5'- CTTGCGCGCACCGGAACTCT -3'/5'- GCCGCTCGAGCTCGTCTTTGG -3' |
| *TaWRKY3* | *TaWRKY3* Full Length | 5'- ACGCTCGACCTCCATCTCAC -3'/5'- GGACTGAGTATGCTGGGCTGTAG -3' |
| *TaWRKY4* | *TaWRKY4* Full Length | 5'- TTCTCTACAATGCTATGGCACAGC -3'/5'- ATCGACATGCCTGAAAGTTGC -3' |
| *TaWRKY5* | *TaWRKY5* Full Length | 5'- GCGTCCGGTCTGATCTAAGCAA -3'/5'- AATGGAGGGGAAAGGGGTAGC -3' |
| *TaWRKY6* | *TaWRKY6* Full Length | 5'- GGGCCATGCTAGGTTCAGACAA -3'/5'- CTGCCGCAGCTTGGTTGTG -3' |
| *TaWRKY7* | *TaWRKY7* Full Length | 5'- CTGGCTGGCATGGCGTCTTC -3'/5'- CGGCGGCGAGGAGCTGTAGT -3' |
| *TaWRKY8* | *TaWRKY8* Full Length | 5'- AGGGGTCAACACTGCATCTTTGT -3'/5'- TGCTTACAAAAAATGCCCACGA -3' |
| *TaWRKY9* | *TaWRKY9* Full Length | 5'- CTCATTTCATATTGAACTCCCTGCTCCT- 3'/5'- TATTGTACACGTGGACCCACATGTAAAC -3' |
| *TaWRKY10* | *TaWRKY10* Full Length | 5'- AGCTCGTCTGTGCAGTGCACTTAT -3'/5'- TCGTGTACATGCATCCGTGAGATT -3' |
| *TaWRKY1* | *TaWRKY1* RT-PCR | 5'- CGTCCTTCTTCTCGTCGGTGA -3'/5'- TCTTCCTTCTCTTGGAGCAGTGG -3' |
| *TaWRKY2* | *TaWRKY2* RT-PCR | 5'- GTAACAGTGACTTCCTCGCCGTA -3'/5'- GGTAGCAGCATCGGTAGTAGCA |
| *TaWRKY3* | *TaWRKY3* RT-PCR | 5'- GTGCTACCCGACGATGAGAGA -3'/5'- TCGCTCCTGTGGTTGTGATGA -3' |
| *TaWRKY4* | *TaWRKY4* RT-PCR | 5'- AAGAGCAGTGAGCATCCAAGGA -3'/5'- GGCAAAGGGTGATTGTGAGAACTC -3' |
| *TaWRKY5* | *TaWRKY5* RT-PCR | 5'- CGGGGGTCGCAAGAGAGTC -3'/ 5'- GGCCGCTTCGATTTGCTTCT -3' |
| *TaWRKY6* | *TaWRKY6* RT-PCR | 5'- CTCCAGCCGTCCCTTCCA -3'/5'- CCAGTTGCACAGCGTCCAC -3' |
| *TaWRKY7* | *TaWRKY7* RT-PCR | 5'- GTCATGGAGGAAGTACGGTCAGAA -3'/5'- ACGGGTTGGACTCGGATACG -3' |
| *TaWRKY8* | *TaWRKY8* RT-PCR | 5'- GTCTCGTCAACGCTGTCCAATG -3'/5'- GGTGGTCGCAGTAGGAATGGTA -3' |
| *TaWRKY9* | *TaWRKY9* RT-PCR | 5'- ACTACTCTCACATCTTGGCGTCTC -3'/5'- TGCTCCTGCTGCTCCTTGAATA -3' |
| *TaWRKY10* | *TaWRKY10* RT-PCR | 5'- TGTACAATTTCGAAGCCGGT -3'/5'- CATGTTCATCGTCTCGCCT -3' |
| *TaActin* | Wheat Actin | 5'- CTTGTATGCCAGCGGTCGAACA -3'/5'- CTCATAATCAAGGGCCACGTA -3' |
