# Supplementary material for: A Wheat WRKY Transcription Factor TaWRKY10 Confers Tolerance to Multiple Abiotic Stresses in Transgenic Tobacco
Source: PLoS One. 2013 Jun 10;8(6):e65120. doi: 10.1371/journal.pone.0065120 (PMC3677898; doi:10.1371/journal.pone.0065120)
Supplement: Table S2 — Primers for TaWRKY10 used in this article. (DOC) [file pone.0065120.s004.doc]

**Table S2 Primers for *TaWRKY10* used in this article.**

| Gene Name | Primer Name/Function | Sequences of 5’- and 3’- Primers |
| --- | --- | --- |
| *TaWRKY10* | Probe of Southern Blot in wheat | 5'- AGCTCGTCTGTGCAGTGCACTTAT -3'/5'- TCGTGTACATGCATCCGTGAGATT -3' |
| *TaWRKY10* | Probe of Southern Blot in tobacco | 5'- ATGGCGGCTTCGCTAGGACT -3'/5'- TCAGTAGGACTCCGAGGAGT -3' |
| *TaWRKY10* | Green Fluorescent Protein | 5'- CCATGGATGGCGGCTTCGCTAGGACT -3'/5'- ACTAGTGTAGGACTCCGAGGAGTGCG -3' |
| *TaWRKY10* | Transcriptional Activation (pBD-WRKY) | 5'- GAATTCATGGCGGCTTCGCTAGGACT -3'/5'- GGATCCTAGGACTCCGAGGAGTGCGCC -3' |
| *TaWRKY10* | Transcriptional Activation (pBD-WRKY-N1) | 5'- GAATTCATGGCGGCTTCGCTAGGACT -3'/5'- GGATCCCCGAGTCCGGAACCCGATC -3' |
| *TaWRKY10* | Transcriptional Activation (pBD-WRKY-N2) | 5'- GAATTCATGGCGGCTTCGCTAGGACT -3'/5'- GGATCCGGGGGTGACGTGGTTGTGGA -3' |
| *TaWRKY10* | Transcriptional Activation (pBD-WRKY-C1) | 5'- GAATTCGATCGGGTTCCGGACTCGGT -3'/5'- GGATCCTAGGACTCCGAGGAGTGCGCC-3' |
| *TaWRKY10* | Transcriptional Activation (pBD-WRKY-C2) | 5'- GAATTCGGCAGCACGTCGTCGAGG -3'/5'- GGATCCTAGGACTCCGAGGAGTGCGCC-3' |
| *TaWRKY10* | Overexpression in Tobacco | 5'- GGATCCATGGCGGCTTCGCTAGGACT -3'/5'- GGATCCTCAGTAGGACTCCGAGGAGT -3' |
| *TaWRKY10* | Polyclonal Antibody | 5'- GGATCCATGGCGGCTTCGCTAGGACT -3'/5'- AAGCTTGTAGGACTCCGAGGAGTGCG -3' |
| *NtUbiquitin* | NtUbiquitin | 5'- TCCAGGACAAGGAGGGTAT -3'/5'- CATCAACAACAGGCAACCTAG -3' |
| *NtERD10c* | NtERD10c | 5'- GGAAGAAGAGAAGGCGGGTGA -3'/5'- GGTCTTTGAGTGATATCCTGGTA -3' |
| *NtSPSA* | NtSPSA | 5'- GAATTCAGGCGCTTCGTTGTCA -3'/5'- ACCCCTAGTTTCTCCAGTGA -3' |
| *NtToblt* | NtToblt | 5'- ACCGGAAGACTGCATGCA -3'/5'- AACCATCCACCAAAGTTTCA -3' |
| *NtSAM* | NtSAM | 5'- CAGACCAATAAACAAGCTTCA -3'/5'- CCTGAAGGACTCTTTCA -3' |
| *NtSOD* | NtSOD | 5'- GACGGACCTTAGCAACAGG -3'/5'- CTGTAAGTAGTATGCATGTTC -3' |
| *NtCAT* | NtCAT | 5'- TGGATCTCATACTGGTCTCA -3'/5'- TTCCATTGTTTCAGTCATTCA -3' |
| *NtGPX* | NtGPX | 5'- GGTTTGCACTCGCTTCAAG -3'/5'- AGTAGTGGCAAAACAGGAAG -3' |
| *NtrbohD* | NtrbohD | 5'- ACCAGCACTGACCAAAGAA -3'/5'- TAGCATCACAACCACAACTA -3' |
