# Supplementary material for: A Wheat WRKY Transcription Factor TaWRKY10 Confers Tolerance to Multiple Abiotic Stresses in Transgenic Tobacco
Source: PLoS One. 2013 Jun 10;8(6):e65120. doi: 10.1371/journal.pone.0065120 (PMC3677898; doi:10.1371/journal.pone.0065120)
Supplement: Table S3 — Characteristics of WRKYs from wheat. (DOC) [file pone.0065120.s005.doc]

**Table S3 Characteristics of WRKYs from wheat**.

| Gene Name | cDNA | | Tigr EST/TC accession No. | Amino Acid  (aa) | N-Terminal | WRKY Domain | Zinc Finger Motif | Sub Group |
| --- | --- | --- | --- | --- | --- | --- | --- | --- |
|  | Full length | Length (bp) |  |  |  |  |  |  |
| TaWRKY1 | Y | 999 | TC395737 | 324 | MAVDLMG | WRKYGQK | C_2_H_2_ | Ⅱ |
| TaWRKY2 | Y | 800 | TC379497 | 212 | MQTQSRL | WRKYGQK | C_2_H_2_ | Ⅱ |
| TaWRKY3 | Y | 848 | TC461590 | 243 | MHYSMSG | WRKYGQK | C_2_H_2_ | Ⅱ |
| TaWRKY4 | Y | 842 | TC387095 | 265 | MAQPSPT | WRKYGQK | C_2_H_2_ | Ⅱ |
| TaWRKY5 | Y | 1137 | TC379684 | 332 | MQRSRGC | WRKYGQK | C_2_HC | Ⅲ |
| TaWRKY6 | N | 995 | TC366284 | 305 | MPLFDSN | WRKYGQK | C_2_H_2_ | Ⅱ |
| TaWRKY7 | Y | 763 | TC445743 | 233 | MASSGGG | WRKYGQK | C_2_HC | Ⅲ |
| TaWRKY8 | Y | 1070 | TC440120 | 297 | MQGVEGR | WRKYGQK | C_2_H_2_ | Ⅱ |
| TaWRKY9 | Y | 1436 | TC369329 | 440 | MSSSTGS | WRKYGQK | C_2_H_2_ | Ⅰ |
| TaWRKY10 | Y | 792 | TC393617 | 222 | MAASLGL | WRKYGKK | C_2_H_2_ | Ⅱ |
