# Supplementary material for: A Wheat WRKY Transcription Factor TaWRKY10 Confers Tolerance to Multiple Abiotic Stresses in Transgenic Tobacco
Source: PLoS One. 2013 Jun 10;8(6):e65120. doi: 10.1371/journal.pone.0065120 (PMC3677898; doi:10.1371/journal.pone.0065120)
Supplement: Table S4 — TaWRKY1 - TaWRKY10 expression patterns in wheat ( Triticicum aestivum cv Chinese Spring) under abiotic stresses. (DOC) [file pone.0065120.s006.doc]

**Table S4 *TaWRKY1*-*TaWRKY10* expression patterns in wheat (*Triticicum aestivum* cv Chinese Spring) under abiotic stress**es.

| Genes | Tigr EST/TC accession No. | Salt | Cold | Drought |
| --- | --- | --- | --- | --- |
| TaWRKY1 | TC395737 | / | + | / |
| TaWRKY2 | TC379497 | / | / | / |
| TaWRKY3 | TC461590 | / | / | / |
| TaWRKY4 | TC387095 | / | - | / |
| TaWRKY5 | TC379684 | / | / | / |
| TaWRKY6 | TC366284 | / | + | + |
| TaWRKY7 | TC445743 | / | / | / |
| TaWRKY8 | TC440120 | / | / | + |
| TaWRKY9 | TC369329 | + | + | - |
| TaWRKY10 | TC393617 | + | + | + |
| The experiments were performed with RT-PCR and repeated at least three times with independent RNA samples and the results were consistent. | | | | |
| + indicates up regulation of gene expressions. | | | | |
| - indicates down regulation of gene expressions. | | | | |
| / indicates no change in gene expressions. | | | | |
