# Supplementary material for: A Wheat WRKY Transcription Factor TaWRKY10 Confers Tolerance to Multiple Abiotic Stresses in Transgenic Tobacco
Source: PLoS One. 2013 Jun 10;8(6):e65120. doi: 10.1371/journal.pone.0065120 (PMC3677898; doi:10.1371/journal.pone.0065120)
Supplement: Table S5 — The GenBank accession numbers of WRKY proteins used for drawing phylogenetic tree. (DOC) [file pone.0065120.s007.doc]

**Table S5 The GenBank accession numbers of WRKY proteins used for drawing phylogenetic tree.**

| Genes | GenBank accession No. |
| --- | --- |
| ZmWRKY67 | EU960054 |
| HvWRKY17 | ABI13383 |
| OsWRKY10 | DAA05075.1 |
| AtWRKY50 | NP_197989.2 |
| AtWRKY34 | NP_194374.1 |
| AtWRKY33 | NP_181381.2 |
| HvWRKY46 | AAQ63880.1 |
| AtWRKY60 | NP_180072.1 |
| AtWRKY18 | NP_567882.1 |
| AtWRKY40 | NP_178199.1 |
| AtWRKY36 | NP_564976.1 |
| AtWRKY9 | NP_176982.1 |
| AtWRKY6 | NP_564792.1 |
| AtWRKY31 | NP_567644.1 |
| AtWRKY17 | NP_565574.1 |
| AtWRKY7 | NP_194155.1 |
| AtWRKY14 | NP_564359.1 |
| AtWRKY35 | NP_181029.1 |
| AtWRKY22 | NP_192034.1 |
| AtWRKY29 | NP_194086.3 |
| AtWRKY46 | NP_182163.1 |
| AtWRKY30 | NP_568439.1 |
| AtWRKY41 | NP_192845.1 |
| AtWRKY53 | NP_194112.1 |
